# Partial IGF-1 deficiency is sufficient to reduce heart contractibility, angiotensin II sensibility, and alter gene expression of structural and functional cardiac proteins

**DOI:** 10.1371/journal.pone.0181760

**Published:** 2017-08-14

**Authors:** José Luis González-Guerra, Inma Castilla-Cortazar, Gabriel A. Aguirre, Úrsula Muñoz, Irene Martín-Estal, Elena Ávila-Gallego, Miriam Granado, Juan E. Puche, Ángel Luis García-Villalón

**Affiliations:** 1 Escuela de Medicina, Tecnológico de Monterrey, Monterrey, México; 2 Fundación de Investigación Hospitales de Madrid, HM Hospitales, Madrid, Spain; 3 Department of Medical Physiology, Faculty of Medicine, University CEU-San Pablo, Madrid, Spain; 4 Department of Physiology, Faculty of Medicine, Universidad Autónoma de Madrid, Madrid, Spain; 5 Department of Cardiology, Hospital Universitario Puerta del Mar, Cádiz, Spain; Max Delbruck Centrum fur Molekulare Medizin Berlin Buch, GERMANY

## Abstract

Circulating levels of IGF-1 may decrease under several circumstances like ageing, metabolic syndrome, and advanced cirrhosis. This reduction is associated with insulin resistance, dyslipidemia, progression to type 2 diabetes, and increased risk for cardiovascular diseases. However, underlying mechanisms between IGF-1 deficiency and cardiovascular disease remain elusive.

The specific aim of the present work was to study whether the partial IGF-1 deficiency influences heart and/or coronary circulation, comparing vasoactive factors before and after of ischemia-reperfusion (I/R). In addition, histology of the heart was performed together with cardiac gene expression for proteins involved in structure and function (extracellular matrix, contractile proteins, active peptides); carried out using microarrays, followed by RT-qPCR confirmation of the three experimental groups. IGF-1 partial deficiency is associated to a reduction in contractility and angiotensin II sensitivity, interstitial fibrosis as well as altered expression pattern of genes involved in extracellular matrix proteins, calcium dynamics, and cardiac structure and function.

Although this work is descriptive, it provides a clear insight of the impact that partial IGF-1 deficiency on the heart and establishes this experimental model as suitable for studying cardiac disease mechanisms and exploring therapeutic options for patients under IGF-1 deficiency conditions.

## Introduction

A large amount of data support that insulin like growth factor 1 (IGF-1) deficiency increases insulin resistance, impairs lipid metabolism, promotes oxidative damage, and dysregulates the GH/IGF-1 axis [1–3]. Interestingly, growth hormone (GH) and insulin act in symphony with IGF-1 to induce a metabolic coordinated response [4–6]. Also, IGF-1 circulating levels decrease with ageing [7]. This reduction is associated with insulin resistance, dyslipidemia, and progression to metabolic syndrome (MetS) establishment, including also liver steatosis, hyper-lipidemia and visceral obesity [2,8–11].

In this context, consistent studies demonstrate the association between IGF-1 deficiency and increased risk for cardiovascular diseases [12–15], such as ischemic heart disease, ischemic stroke, and congestive heart failure in the elderly [14,16,17], as well as a worse recovery prognosis after an acute myocardial infarction [18]. However, the underlying mechanisms between IGF-1 deficiency and cardiovascular disease are poorly understood. In order to gain more insight into these mechanisms, a previously described experimental model of partial IGF-1 deficiency was used [19]. We included three groups of mice (5 ± 2 months old): untreated heterozygous (Hz, *Igf*^*+/-*^*)* with partial IGF-1 deficiency; heterozygous mice treated with low doses of IGF-1; and wild type (Wt, *Igf*^*+/+*^*)* mice used as control group.

The specific aim of the present work was to study whether partial IGF-1 deficiency influences heart and/or coronary circulation, comparing vasoactive factors before and after of ischemia-reperfusion (I/R). In addition, histology of the heart was performed together with cardiac gene expression for proteins involved in structure and function (extracellular matrix, contractile proteins, active peptides); carried out using microarrays, followed by RT-qPCR confirmation of the three experimental groups.

## Materials and methods

### Animals and experimental design

The experimental model was established and characterized as previously reported [19]. Briefly, IGF-1 heterozygous mice were obtained by cross-breeding transgenic mice, line MF1 and 129SV^*Igf1/tm1Arge*^ [20]. Animal genotype determination was performed by PCR analysis (Applied Biosystems, 2720 Thermal Cycler, Spain). DNA was extracted from a piece of tail and specific primers were used to identify both *Igf1* and *Neo* genes (Extract-N-Amp TM Tissue PCR KIT Sigma, USA). Animals were housed in cages in a room with a 12-hour light/dark cycle, constant humidity (50–55%) and temperature (20–22°C). Food (Teklad Global 18% protein rodent diet, Harlan Laboratories, Spain) and water were given *ad libitum*. All experimental procedures were performed in compliance with The Guiding Principles for Research Involving Animals and approved by the Bioethical Committee from our institutions (Tecnologico de Monterrrey and San Pablo-CEU University).

As aforementioned, in the experimental design, three groups of 5 ± 2-months-old male mice were included: Control wildtype group, (Wt, *Igf1*^*+/+*^*);* heterozygous group (Hz, *Igf1*^*+/-*^*)* with partial IGF-1 deficiency; and Hz + IGF-1 group, heterozygous (*Igf1*^*+/-*^*) mice* receiving 2μg/100g/day of recombinant human IGF-1 (Chiron Corporation, USA) for 10 days. Wt and Hz groups received also received the same vehicle in which IGF-1 was administered. Total mice used for the study was 60 animals, n = 20 per group.

On day 11, mice were weighed, blood was obtained from submandibular vein and thereafter 10 animals per group were sacrificed by cervical dislocation, 5 per group were anesthetized and used for cardiac functional studies, and the remaining 5 for histopathological studies. For the former, the heart was carefully dissected out, weighed (Denver Instrument, Germany), and stored in RNAlater (Qiagen-Izasa, Spain) at -80°C for microarray and PCR analyses. Handling of animals for functional studies are described below. For the latter (histological studies), hearts were included in 4% paraformaldehyde for histological preparations.

IGF-1 serum concentration was determined before and after (day 0 and day 11) the protocol. Serum was isolated from blood obtained from submandibular vein. A commercial enzyme-linked immunosorbent assay (ELISA) was used for determinations (Chiron Corporation, USA), following the manufacturer’s instructions, read in a Varioskan spectrophotometer (Thermo Scientific, Spain), and interpreted using SkanIt software (Fisher Scientific, Spain).

### Histopathological studies

Samples of heart for histopathological analysis (n = 5 animals per group) were fixed in 4% paraformaldehyde diluted in PBS solution for 24h. Once they were properly fixed, they were included in ethanol (70%). Samples were embedded in paraffin using the automated equipment (Leica TP 1020, Leica, Switzerland). Longitudinal sections 4-μm-thick were cut using a microtome (Reichert Jung Biocut 2030, Leica, Switzerland) and subsequently stained with either hematoxylin–eosin or Masson’s trichrome. After histological preparations were scanned (Leica, biosystems scanner), two different observers analyzed them using Aperio Image program and estimated the length and thickness of cardiac fibers and collagen depositions, both perivascular ant inter-fibrillary, for the whole preparation.

### Heart perfusion

Five animals from each group were used for this assay. As previously described [21,22], hearts were removed from the mice under anesthesia with i.p. sodium pentobarbital (200 mg/kg) and following i.v. injection of heparin (1000 UI). Next, the ascending aorta was cannulated and the heart was subjected to retrograde perfusion with Krebs- Henseleit buffer (115 mMNaCl, 4.6 mMKCl, 1.2 mMKH2PO4, 1.2 mM MgSO4, 2.5 mM CaCl2, 25 mM NaHCO3 and 11 mM glucose) equilibrated with 95% oxygen and 5% carbon dioxide to a pH of 7.3–7.4. Perfusion was initiated in a non-recirculating Langendorff heart perfusion apparatus at a constant flow rate of 11–15 ml/min to provide a basal perfusion pressure of approximately 70 mmHg. Both the perfusion solution and the heart were maintained at 37°C. Coronary perfusion pressure was measured through a lateral connection in the perfusion cannula and left ventricular pressure was measured using a latex balloon inflated to a diastolic pressure of 5–10 mmHg, both connected to Statham transducers (Statham Instruments, Los Angeles, California). Left ventricular developed pressure (systolic left ventricular pressure minus diastolic left ventricular pressure), the first derivate of the left ventricular pressure curve (dP/dt) and heart rate (HR) were calculated from the left ventricular pressure curve. These parameters were recorded on a computer using Chart 5 v5.4.1 software and the PowerLab/8SP data acquisition system (ADInstruments, Colorado Springs, Colorado).

After a 15min equilibration period with constant flow perfusion, the hearts were exposed to global zero-flow ischemia for 30min and re-perfused for 15min at the same flow rate used before ischemia. The duration of ischemia and reperfusion were chosen on the basis of previous studies demonstrating decreases in the endothelium-dependent coronary relaxation without alteration of endothelium-independent coronary relaxation [23,24]. The control hearts were perfused during a similar total time (60min) at constant flow without ischemia. After I/R or perfusion during 60min the coronary vasoconstriction to angiotensin II or the vasodilatation to bradykinin was recorded. Angiotensin II was injected into the perfusion cannula with an infusion pump over 3min at a constant rate to reach a final concentration of 10212– 1027M. The relaxation to bradykinin was recorded after precontracting the coronary arteries with the thromboxane A2 analogue U46619. First, 1028M U46619 was added to the perfusion solution and the concentration was increased progressively until a contractile tone of, 120–140mmHg was obtained. The concentrations of U46619 required to achieve this effect was 161028 to 361028M in control conditions and 561028 to 261027M after I/R. When the contractile tone reached a stable level, bradykinin was injected into the perfusion cannula over 2min at a constant rate to reach a final concentration of 1029–1026M). As the experiments were performed at a constant flow rate, the coronary perfusion pressure provides a measure of the perfusion resistance and characterizes the contraction or relaxation of the coronary arteries.

### Gene expression studies

#### Microarray analysis

As previously reported [1,25,26], heart mRNA was isolated from animals belonging to the three experimental groups in accordance with the protocol outlined in RNAqueousH-Micro Kit (Ambion, USA). Technical procedures for microarray analysis, including quality control of mRNA, labeling, hybridization and scanning of the arrays were performed according to standard operating procedures for Affymetrix protocols (GeneChipH Expression Analysis Manual, Affymetrix, USA). The mRNAs were profiled using Affymetrix HT MG-430. The array signals were normalized using Robust Multichip Averages [27] and batch-effects of the three replicates were corrected using ComBat [28]. Differentially expressed genes between samples were selected using FDR-corrected p-value of 0.01 (p value of <0.05). Data is available at the EMBL Array Express repository under the accession number E-MTAB-5791.

#### Total RNA extraction, reverse transcription and quantitative real time polymerase chain reactions (RT-qPCR)

The hearts belonging to this group were cryopreserved in RNAlater (Qiagen-Izasa, Spain) as previously mentioned. The day performing PCR determinations heart samples were homogenized with TRIzol reagent (Invitrogen, UK) by Tissue Lyser LT (Qiagen-Izasa, Spain) and RNA was extracted and purified using the RNeasy Mini Kit (Qiagen), including digestion with RNase-free DNase, following the manufacturer’s instructions. RNA purity was checked using Nanodrop A260:A280 ratio (Thermo Fisher Scientific, CA, USA) and fragment integrity analysed with Bioanalyzer 2100 (Agilent Technologies Inc., USA). Purified RNA was then converted to cDNA by using the RNA-to-DNA EcoDryTM Premix (Clonetech Labs, USA) for RT-qPCR assays. RT-qPCR assays were performed in a 3100 Avant Genetic Analyzer (Applied Biosystems Hispania, Spain). The thermal profile consisted of an initial 5min melting step at 95°C followed by 40 cycles at 95°C for 10s and 60°C for 60s. Specific Taqman® probes for the selected genes were supplied by Applied Biosytems.

The relative mRNA levels of the genes of interest were normalized to *Tbp* expression using the well-stablished simplified comparative threshold cycle delta, cycle threshold (CT) method [2^-(ΔCT gene of interest- ΔCT actin)^] [29]. *Tbp* was selected for endogenous control after carefully analyzing 12 housekeeping genes (*Actb*, *B2m*, *Gapdh*, *Gusb*, *Hsp90ab1*, *Ldha*, *Pgk1*, *Ppih*, *Sdha*, *Tbp*, *Tfrc*, *and Ubc*). Only 2 out of these 12 vary their expression when comparing Hz with Wt Controls. We assayed the others 10 genes and selected the one, which showed the most stable and reproducible values, which was *Tbp*, a very stable gene coding for a transcription factor that binds the TATA box.

### Statistical analysis

All data represent mean ± SEM. Statistical analysis was performed on SPSS 20 (Statistical Package for Social Sciences, USA). Significance was estimated by the Kruskal-Wallis ANOVA followed by a post-hoc test for distribution-free multiple comparisons (Bonferroni). Correlation was analyzed by Spearman test or "r of Pearson". Differences were considered significant at a level of p<0.05.

## Results

### IGF-1 circulating levels and body and heart weights

In accordance with previous series, Hz mice showed a significant reduction of serum circulating IGF-1 levels compared to Wt controls (Hz = 510.21±56.12ng/ml vs. Wt = 679.18 ±53.14ng/ml, p<0.01). The subcutaneous administration of IGF-1 at low doses, during 10 days normalized circulating levels of this hormone (Hz+IGF-1 = 667.72±86.69ng/ml, p<0.01 vs. untreated Hz group; p = ns vs. Wt group).

As expected, IGF-1 deficient mice had a significant reduction in absolute body weight (Hz = 31.60±1.11g vs. Wt = 38.83±2.25g, p<0.01), which IGF-1 therapy restored to normal values (Hz+IGF-1 = 38.17±1.52g, p = ns vs. Wt).

No differences between groups were found in absolute heart weight. However, when the absolute heart weight was referred to body weight, both groups of Hz mice presented significant increase of relative heart weight (heart weight (mg)/body weight (g), Hz = 5.2±0.16, Hz+IGF-1 = 5.3±0.14 and Wt = 4.8±0.17, p<0.05 both Hz group vs. Wt group).

### Hemodynamic parameters in the perfused hearts

Table 1 summaries the hemodynamic values in perfused hearts from controls (Wt, *Igf*^*+/+*^*)*, untreated mice with IGF-1 deficiency (Hz, *Igf*^*+/-*^*)* and Hz mice treated with low doses of IGF-1. No significant differences were found before I/R for HR, coronary perfusion pressure, left developed intraventricular pressure, and maximal dP/dt.

**Table 1:** Hemodynamic values in perfused hearts from controls, mice with IGF-1 deficiency (Hz) and Hz treated with IGF-1 therapy.

**Results before ischemia**				
**Experimental group**	**Heart rate (beats/min)**	**Coronary perfusion pressure (mmHg)**	**Left IV developed pressure (mmHg)**	**dP/dt (mmHg/s)**
Controls (Wt) (*Igf*^*+/+*^)	315 ± 31	59 ± 10	47 ± 7	1748 ± 315
Untreated IGF-1 deficient mice. (Hz) (*Igf*^*+/-*^)	298 ± 11	63 ± 15	31 ± 7	1256 ± 270
Deficient mice treated with IGF-1 (Hz + IGF-1)	285 ± 31	49 ± 8	36 ± 6	1465 ± 259
**Results after ischemia-reperfusion**				
**Experimental group**	**Heart rate (beats/min)**	**Coronary perfusion pressure (mmHg)**	**Left IV developed pressure (mmHg)**	**dP/dt (mmHg/s)**
Controls (Wt) (*Igf*^*+/+*^)	188 ± 17	41 ± 6	25 ± 8	644 ± 239
Untreated IGF-1 deficient mice. (Hz) (*Igf*^*+/-*^)	264 ± 37 ^#^	35 ± 4	32 ± 10	1140 ± 363^#^
Deficient mice treated with IGF-1 (Hz + IGF-1)	160 ± 42 ^&^	49 ± 9	26 ± 6	818 ± 259^&^

n = 5 animals per group.

# p<0.05 Hz vs. control group

& p<0.05 Hz+IGF-1 vs. untreated Hz group.

**Fig 1:**
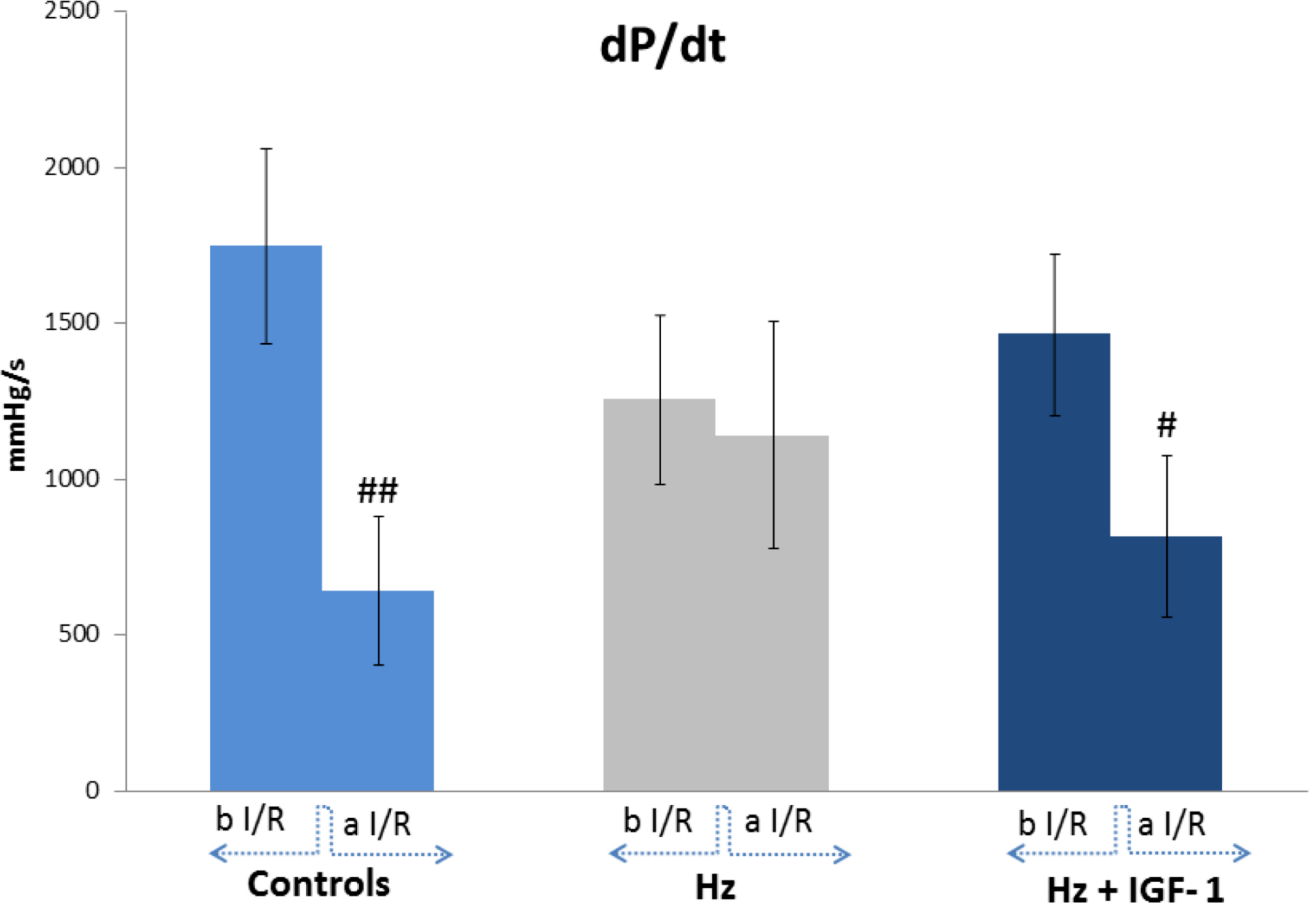
Hemodynamic values in perfused hearts from the three experimental groups. dP/dt (mmHg/s), expressing left ventricular contractility, before and after I/R. Before I/R IGF-1 deficient mice showed a reduction of dP/dt but did not reach statistical significance, whereas Hz+IGF-1 mice presented quite similar values to controls (Wt mice). After, I/R, hearts from controls showed a significant reduction in contractility (dP/dt) and similar results were observed in hearts from Hz+IGF-1 group. However, no response after I/R was found in untreated IGF-1 deficient mice (p = ns). ## p<0.01 controls after I/R vs before I/R; # p<0.05 Hz+IGF-1 after I/R vs the same group before I/R (n = 5 each group).

I/R induced a significant increase in HR and dP/dt in untreated IGF-1 deficient mice (Hz group). Interestingly, IGF-1 therapy restores these parameters to similar values those found in Wt controls (Hz-IGF-1 vs. Wt, p = ns). dP/dt ratio was used as a marker of ventricular contractility. As mentioned before (Table 1), I/R in untreated Hz mice showed a reduction of dP/dt but did not reached statistical significance, whereas Hz+IGF-1 group presented quite similar values to those found in Wt controls. After I/R, hearts from controls showed, as expected, a significant reduction in contractility (dP/dt) and a similar result was observed in hearts from Hz+IGF-1 group. However, no differences between before and after (I/R) were found in hearts from untreated IGF-1 deficient mice (Hz). These results are summarized in Fig 1.

### Coronary vasoconstriction to angiotensin II (Ang II)

**Fig 2:**
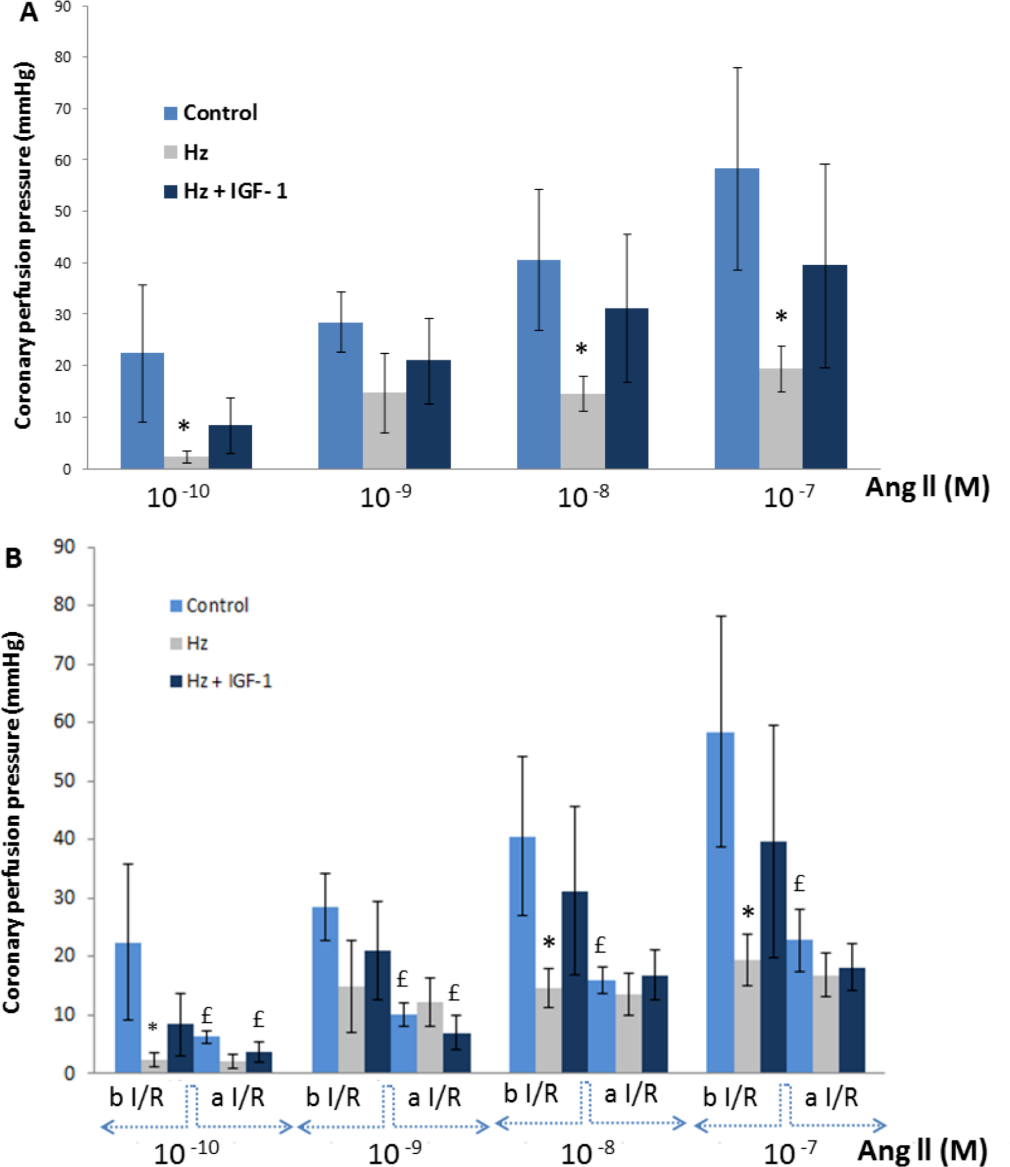
Coronary vasoconstriction to Ang II. **(A)** Coronary perfusion pressure (mmHg) in hearts from the three experimental groups, at different concentrations (10^−10^ to 10^−7^) of Ang II before Ischemia/Reperfusion: Ang II induced vasoconstriction dose-dependent on perfused hearts from controls and Hz+IGF-1, increasing coronary perfusion pressure. However, no effect was observed after Ang II injection in hearts from Hz group. **(B)** Coronary perfusion pressure after I/R: vasoconstriction to Ang II was reduced both in controls and Hz+IGF-1 group. But no response was observed in untreated Hz group, showing a quite total insensibility to AngII (n = 5 per group). * p<0.05 Hz vs Controls; £ p<0.05 after vs. before I/R in the same group (control or Hz+IGF-1).

In the perfused hearts from Wt controls and Hz mice treated with IGF-1, injection of Ang II induced vasoconstriction in a dose dependent manner, increasing coronary pressure (Fig 2A). However, such vasoconstriction was absent in perfused hearts belonging to IGF-1 deficient mice (untreated Hz group) at all concentrations except for 10^-7^M which shows a moderate increase in pressure (Fig 2A). In addition, after I/R, the vasoconstriction achieved by Ang II was reduced in hearts from Wt controls (Fig 2B) and in hearts from IGF-1 treated Hz mice (Hz+IGF-1). However, in perfused hearts from untreated Hz group it was found a very high insensibility to Ang II, without any appreciable vasoconstrictor effect.

### Coronary vasodilatation to bradykinin

**Fig 3:**
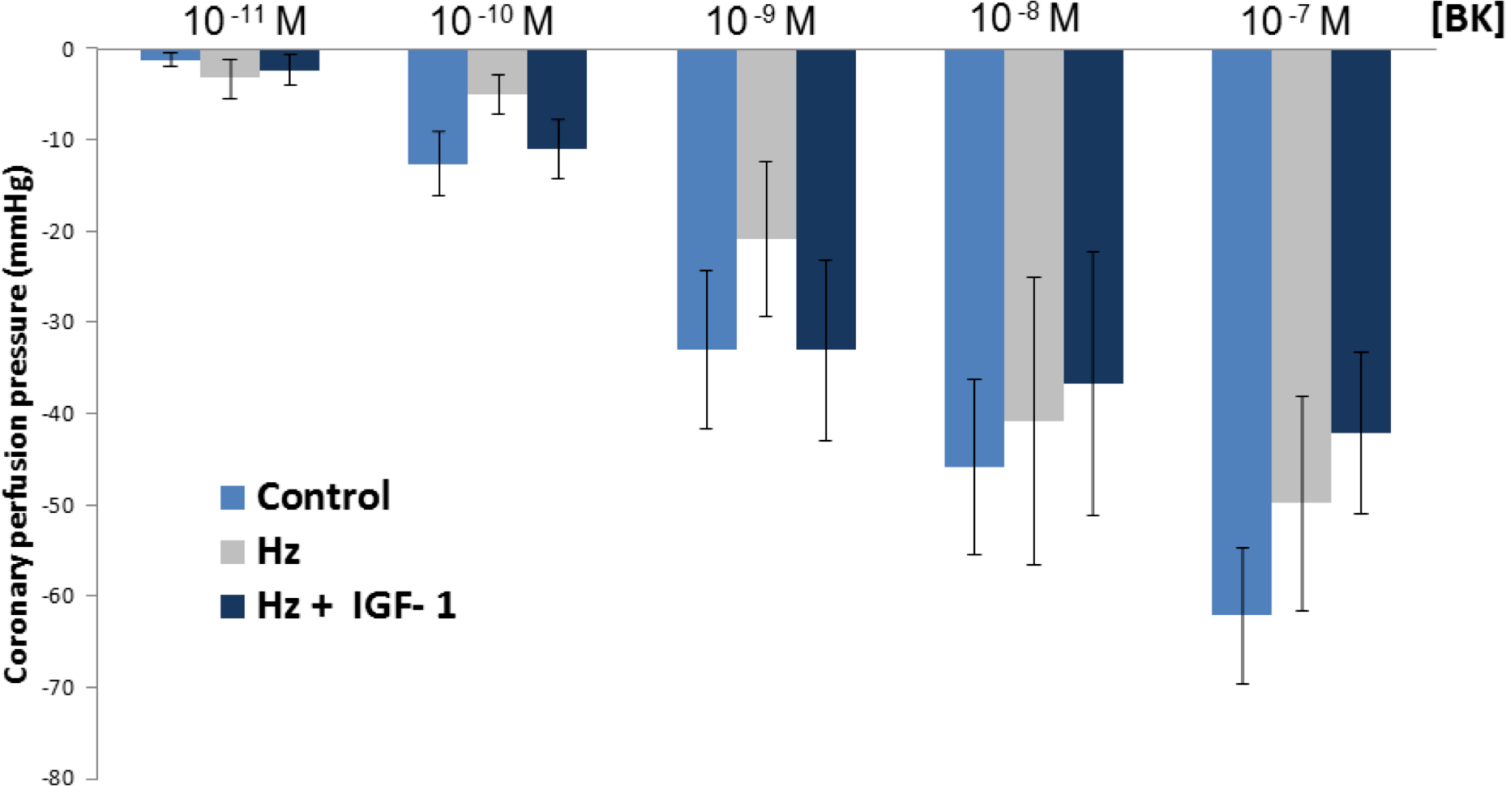
Coronary vasodilatation to bradykinin: no significant differences were found between the three experimental groups in bradykinin-induced-vasodilatation (n = 5 per group).

Fig 3 summarizes results obtained from perfused hearts of three experimental groups to different bradykinin concentrations. No significant differences were found between groups in the vasodilatation induced by bradykinin. Vasodilatation to bradykinin is considered as an endothelial dependent response. Results after I/R were deleted as considered irrelevant.

As is shown in Fig 3, only at 10^-9^M bradykinin was observed a similar trend that the one found in response to the biphasic effect of Ang II, where untreated Hz mice showed a diminished vasodilatation, in this case without reaching statistical significance.

### Histopathological analysis

**Fig 4:**
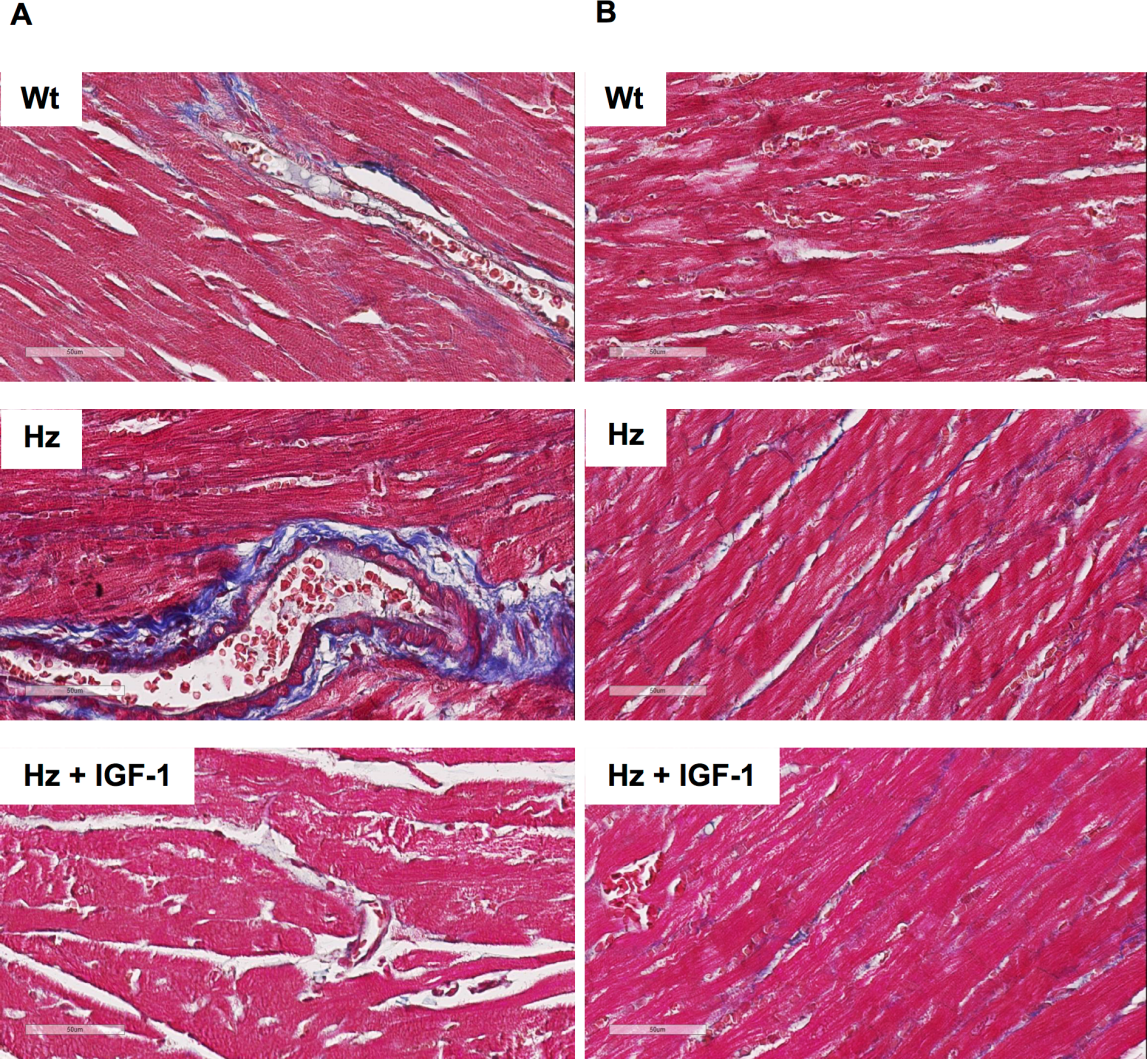
Histopathological study (n = 5). **(A)** Pictures from the three experimental groups showing a remarkable perivascular fibrosis in hearts from Hz group that was not found in controls and Hz+IGF-1 group. **(B)** Interstitial fibrosis, more evident in untreated Hz group as compared to Wt and Hz+IGF-1 experimental groups.

The histopathological analysis study (Masson’s Trichrome) showed increased collagen deposition in hearts from untreated IGF-1 deficient mice (Hz group). Fibrosis was observed around vessels and in-between cardiomyocytes. No significant differences were found in the length (data not shown) and thickness of the muscle fibers (Wt 14.66±3.9μm; Hz 16.97±3.15μm; Hz+IGF-1 17.81±2.50μm, p = ns). Fig 4 shows the histopathological findings in the three experimental groups. In addition, focus of interstitial (sub-endocardial or sub-epicardial) fibrosis were formed in 3 of the 5 animals belonging to the untreated Hz group, and in 1 out of 5 from the IGF-1-treated Hz group. No fibrotic focuses were found in Wt mice.

### Heart gene expression analysis

Microarray results analysis revealed, from a total of 45,078 scanned genes, 582 were down-regulated (considered when expression fold-change was -1.5) when confronting expressions between Hz and Wt groups. Also, 296 genes were up-regulated (fold-change over +1.5) between those groups. On the other hand, 645 genes were underexpressed and 371 were over-expressed when comparing Hz+IGF1-1 vs. untreated Hz group.

#### IGF-1 related gene expression

**Fig 5:**
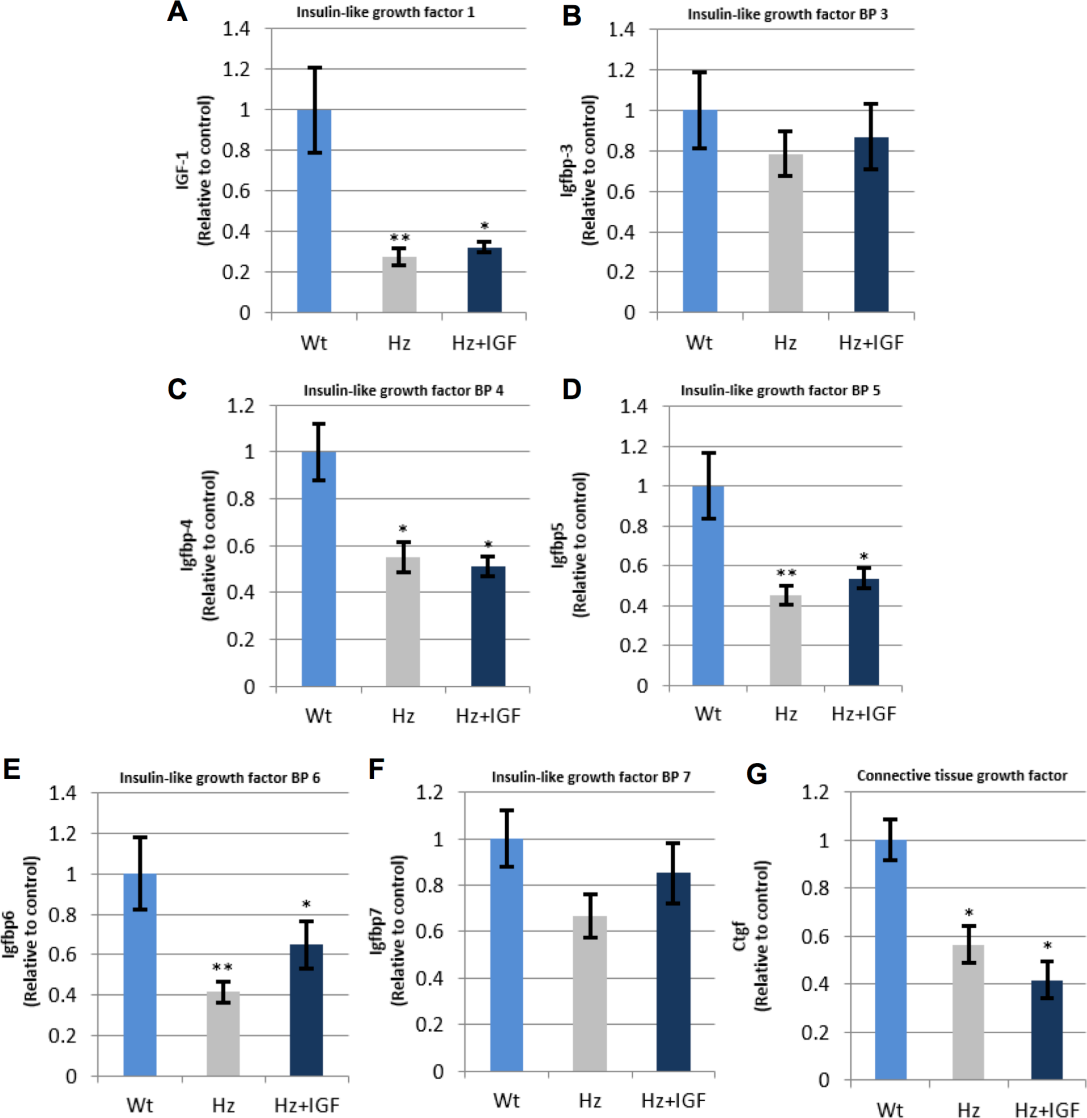
qPCR-RT measurement of RNA (cDNA) expression of genes encoding proteins closely related to IGF-1 function. *p<0.05, ** p 0.01 vs. controls (Wt group). **(A)** *Igf1;* **(B), (C), (D), (E), (F)** *Igfbp3*, *4*, *5*, and, 7, respectively; **(G)** *Ctgf/Igfbp8*. n = 10, each group.

Among the genes with an altered expression, we first focused on those encoding proteins closely related to IGF-1 function (Table 2). RT- qPCR was performed to confirm changes over ±1.5 fold-change, such as hypo-expression of *Igf-1* and *Igfbp 3*, *4*, *5*, *6*, *7* and *8* as compared to Wt controls. Little effects were induced by IGF-1 replacement therapy in the expression of these genes (Fig 5).

**Table 2:** Altered expression of genes encoding proteins closely related to IGF1 physiology.

Gene name		Untreated Hz (*Hz*. *Igf*^*+/-*^*)* vs. Controls (WT. *Igf*^*+/+*^) (Fold Change)	Hz + *IGF1* vs. Untreated Hz (Fold Change)
**insulin-like growth factor 1**	***Igf1***	***-1*.*72 (p≤0*.*05)***	*1*.*15* (p≤0.01)
insulin-like growth factor I receptor	*Igf1r*	1.01 (p = 0.64)	-1.39(p≤0.01)
**insulin-like growth factor 2**	***Igf2***	***-3*.*75 (p≤0*.*01)***	1.07(p = 0.48)
insulin-like growth factor 2 receptor	*Igf2r*	1.20 (p≤0.05)	-1.31(p≤0.05)
insulin-like growth factor binding protein 1	*Igfbp1*	1.00 (p = 0.95)	1.12(p = 0.21)
insulin-like growth factor binding protein 2	*Igfbp2*	1.49 (p≤0.01)	1.09(p = 0.08)
**insulin-like growth factor binding protein 3**	***Igfbp3***	***-2*.*25 (p≤0*.*01)***	1.01 (p = 0.97)
**insulin-like growth factor binding protein 4**	***Igfbp4***	***-1*.*88 (p≤0*.*01)***	-1.26(p≤0.05)
**insulin-like growth factor binding protein 5**	***Igfbp5***	***-1*.*79 (p≤0*.*001)***	***-1*.*66(p≤0*.*01)***
**insulin-like growth factor binding protein 6**	***Igfbp6***	***-2*.*09(p≤0*.*001)***	***1*.*96 (p≤0*.*01)***
**connective tissue growth factor**	***Ctgf***	***-1*.*65 (p≤0*.*001)***	***-1*.*83(p = 0*.*38)***
gremlin 1	*Grem1*	-1.26(p = 0.09)	-1.02(p = 0.23)

#### Expression of genes encoding proteins involved in heart structure, function and pathology

**Fig 6:**
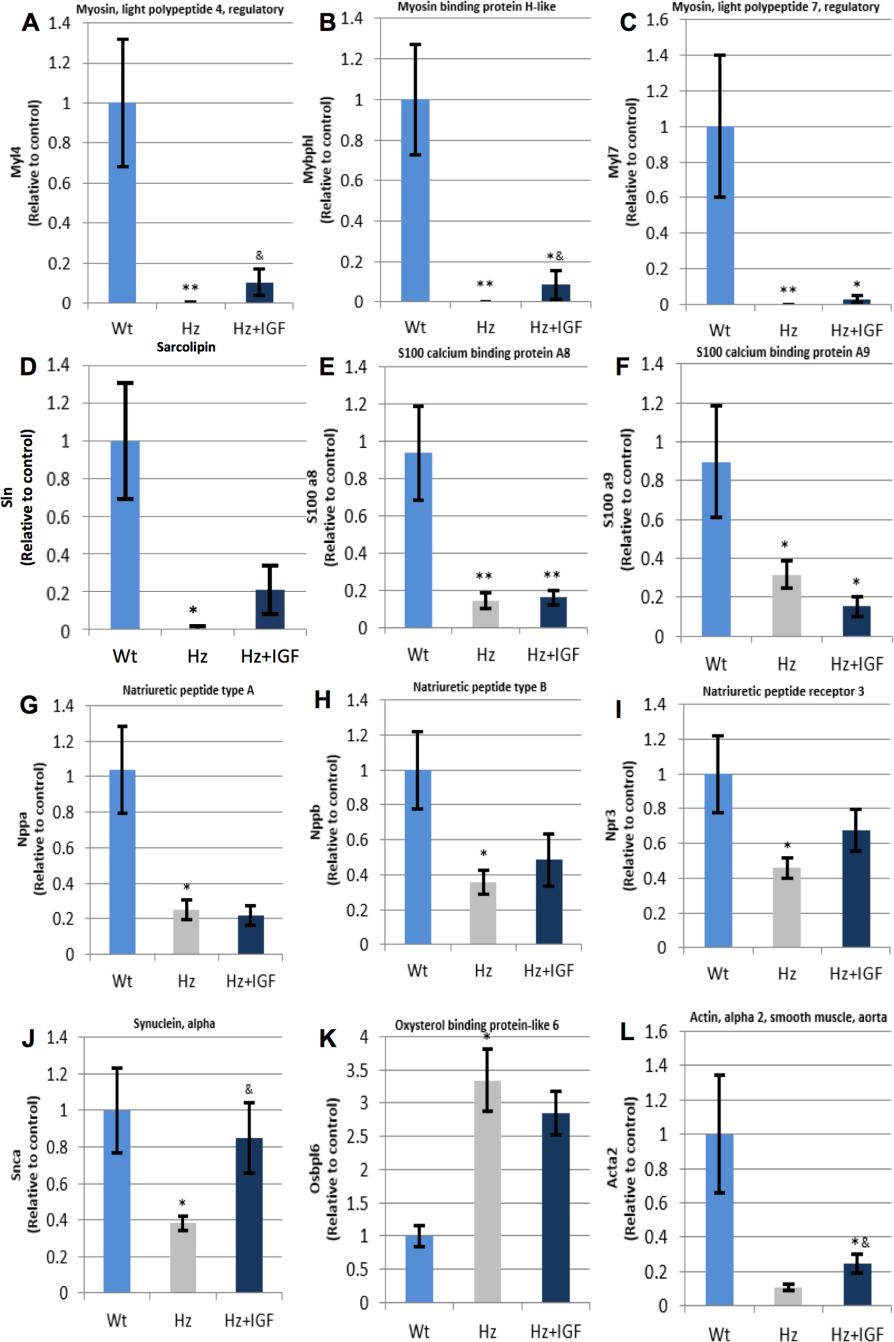
qPCR-RT measurement of RNA (cDNA) expression for coded proteins involved in heart structure, function, and pathology. **(C), (E), (F),** and **(G)** show low expression in both groups of IGF-1 deficient mice for *Myl7*, *S100a8*, *S100a9*, *Nppa*, respectively; **(H), (I), (J),** and **(L)** depict a reduced expression among the untreated Hz group and some recovering with the substitutive treatment (Hz+IGF-1) for *Nppb*, *Npr3*, *Snca*, *Acta2*, respectively; **(K)** shows a marked genetic overexpression for *Osbpl6* in both Hz group of animals. *p<0.05, ** p 0.01 vs. controls (Wt group); & p<0.05 Hz+IGF-1 group vs untreated Hz mice (n = 10 per group).

The most relevant findings were found in genes coding for contractile proteins, calcium dynamic and function, natriuretic peptides type A and B and their receptors, synuclein, among others (Table 3). Of interest, untreated Hz mice showed a noticeable down-regulation for most of these genes. When confirming expression changes by RT-qPCR, results from the treated Hz group were not as expected. Results are included in Fig 6.

**Table 3:** Expression of genes encoding proteins involved in heart structure, function, and pathology.

Gene name		Untreated Hz (*Hz*. *Igf*^*+/-*^*)* vs. Controls (WT. *Igf*^*+/+*^) (Fold Change)	Hz + *IGF1* vs. Untreated Hz (Fold Change)
**gap junction protein, alpha 1**	***Gja1***	1.12 (p = 0.43)	***1*.*73(p≤0*.*05)***
reticulon 4	*Rtn4*	1.21(p = 0.11)	1.01(p = 0.78)
troponin I cardiac 3	*Tnni3*	1.03(p = 0.63)	1.02(p = 0.70)
troponin T2 cardiac	*Tnnt2*	1.01(p = 0.88)	1.04(p≤0.05)
actin, alpha, cardiac muscle 1	*Actc1*	1.03(p = 0.14)	1.01(p = 0.66)
myosin light polypeptide 7 regulatory	***Myl7***	***-229*.*49(p≤0*.*0001)***	1.12(p≤0.001)
myosin light polypeptide 4 regulatory	***Myl4***	***-146*.*94(p≤0*.*0001)***	-1.19(p = 0.75)
myosin binding protein H-like	***Mybphl***	**-16.12(p≤0.0001)**	-1.2(p≤0.01)
myosin heavy polypeptide 6 cardiac muscle alpha	*Myh6*	1.13(p = 0.20)	-1.01(p = 0.60)
nebulette	*Nebl*	-1.06(p = 0.38)	-1.48(p = 0.06)
periostin osteoblast specific factor	***Postn***	-1.05 (p = 0.21)	***-1*.*97(p≤0*.*001)***
tubulin beta 1 class VI	***Tubb1***	***-1*.*63(p≤0*.*01)***	-1.05(p = 0.11)
phospholamban	*Pln*	1.02(p = 0.68)	1.03(p = 0.75)
sarcolipin	***Sln***	***-159*.*33(p≤0*.*0001)***	***1*.*09(p = 0*.*12)***
S100 calcium binding protein A1	*S100a1*	1.01(p = 0.58)	1.01(p = 0.56)
S100 calcium binding protein A6 (calcyclin)	*S100a6*	1.27(p = 0.35)	-1.13(p = 0.52)
S100 calcium binding protein A8 (calgranulin A)	***S100a8***	**-8.86(p≤0.01)**	-1.24(p≤0.01)
S100 calcium binding protein A9 (calgranulin B)	***S100a9***	***-6*.*81*(p≤0.001)**	1.04(p = 0.19)
ryanodine receptor 2 cardiac	*Ryr2*	1.14(p≤0.05)	1.14(p≤0.001)
calsequestrin 2	*Casq2*	1.05(p = 0.15)	1.04(p = 0.15)
triadin	*Trdn*	-1.15(p = 0.60)	1.36(p = 0.39)
calcium channel. voltage-dependent L type alpha 1C subunit	*Cacna1c*	1.37(p≤0.05)	1.27(p≤0.001)
junctional sarcoplasmic reticulum protein 1	*Jsrp1*	1.08(p = 0.40)	-1.03(p = 0.72)
bridging integrator 1	*Bin1*	1.02(p = 0.98)	-1.03(p = 0.99)
natriuretic peptide type A	***Nppa***	***-2*.*17(p≤0*.*0001)***	-1.67 (p≤0.05)
natriuretic peptide type B	***Nppb***	***-1*.*57(p≤0*.*05)***	1.35(p≤0.01)
natriuretic peptide receptor 1	***Npr1***	***1*.*13(p = 0*.*69)***	1.05(p = 0.84)
natriuretic peptide receptor 3	***Npr3***	***-2*.*68(p≤0*.*01)***	-1.77(p≤0.01)
synuclein. alpha	***Snca***	***-3*.*34(p≤0*.*0001)***	-1.52(p≤0.001)
pro-platelet basic protein	***Ppbp***	***-3*.*85(p≤0*.*01)***	1.05(p = 0.61)
actin alpha 2 smooth muscle aorta	***Acta2 (a-SMA)***	***-2*.*2 (p≤0*.*01)***	-1.26 (p≤0.05)
oxysterol binding protein-like 6	***osbpl6***	***5*.*01(p≤0*.*01)***	-2.23(p≤0.01)
apolipoprotein A-I	***Apa1***	***-5*.*36(p≤0*.*01)***	-1.03(p = 0.54)
alpha-2-HS-glycoprotein	***Ahsg***	***-4*.*21(p≤0*.*01)***	-1.35(p≤0.05)

#### Expression of genes coding for proteins implicated in inflammation and extracellular matrix formation in normal and pathological conditions

**Fig 7:**
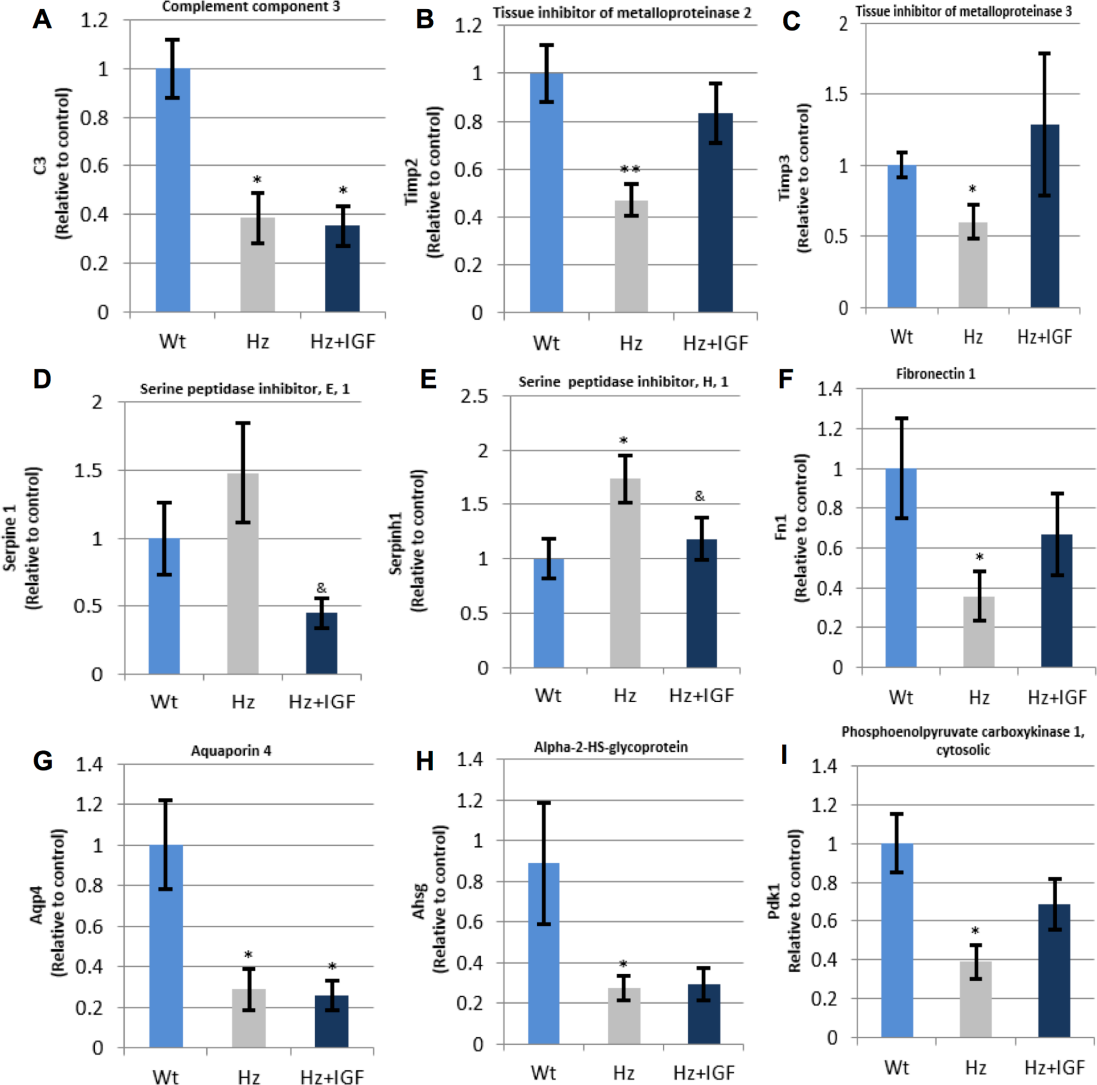
qPCR-RT measurement of RNA (cDNA) expression for coded proteins involved in inflammation, extracellular matrix regulation, and heart metabolism. **(B), (C), (F),** and **(I)** represent those genes (*Timp2*, *Timp3*, *Fn1*, *Pdk1*, respectively) whose expression is downregulated by IGF-1 deficiency and recovered by substitutive treatment; **(A), (G),** and **(H)** refer to those genes (*C3*, *Aqp4*, *Ahsg*, respectively) underexpressed in both Hz groups; **(D)** shows that *Serpine1* was overexpressed in Hz animals and contrarily underexpressed when substitutive treatment was applied; and **(E)** illustrates how *Serpinh1* is upregulated by IGF-1 deficiency and how IGF-1 substitutive therapy normalizes its expression (n = 10 animals per group). *p<0.05, ** p 0.01 vs. controls (Wt group); & p<0.05 Hz+IGF-1 group vs. untreated Hz mice.

Table 4 summarizes data for gene expression related to inflammation processes and extracellular matrix regulation. The most relevant findings are included in Fig 7 showing gene expression confirmed by qPCR-RT.

**Table 4:** Expression of genes encoding proteins involved on inflammation, extracellular matrix regulation, and in heart metabolism.

Gene name		Untreated Hz (*Hz*. *Igf*^*+/-*^*)* vs. Controls (WT. *Igf*^*+/+*^) (Fold Change)	Hz + *IGF1* vs. Untreated Hz (Fold Change)
**complement component 3**	***C3***	-1.06(p≤0.05)	***-1*.*88(p = 0*.*06)***
histone deacetylase 5	*Hdac5*	1.12(p = 0.21)	-1.01(p = 0.32)
Angiotensinogen	*Agt*	-1.17(p≤0.05)	1.04(p = 0.11)
chemokine (C-C motif) ligand 11	*Ccl11 (Eotaxin)*	-1.00 (p = 0.99)	-1.24(p≤0.05)
chemokine (C-C motif) ligand 2	*Ccl2 (MCP-1)*	-1.27(p≤0.05)	-1.04(p = 0.97)
chemokine (C-C motif) ligand 3	*Ccl3(MIP-1a)*	-1.06(p = 0.21)	-1.21(p = 0.53)
interleukin 13	*Il13*	1.06(p = 0.27)	-1.09(p = 0.33)
interleukin 13 receptor alpha 2	*Il13ra2*	-1.1 (p = 0.13)	1.07(p = 0.42)
interleukin 4 receptor alpha	*Il4ra*	-1.2 (p = 0.15)	-1.05(p = 0.56)
interleukin 5	*Il5*	-1.05(p = 0.14)	-1.01(p = 0.94)
snail family zinc finger 1	*Snai1*	-1.28(p = 0.13)	1.03 (p = 0.61)
lysyl oxidase	*Lox*	1.04(p = 0.28)	1.1(p = 0.07)
matrix metallopeptidase 13	*Mmp13*	-1.08(p = 0.13)	1.13(p = 0.55)
matrix metallopeptidase 14 (membrane-inserted)	*Mmp14*	1.17(p = 0.39)	1.56 (p = 0.07)
matrix metallopeptidase 2	*Mmp2*	1.08(p≤0.05)	1.09(p≤0.05)
matrix metallopeptidase 3	*Mmp3*	1.11(p≤0.05)	-1.32(p≤0.05)
matrix metallopeptidase 8	*Mmp8*	-1.27(p≤0.001)	1.02(p = 0.15)
matrix metallopeptidase 9	*Mmp9*	1.08(p = 0.30)	-1.01(p = 0.85)
plasminogen activator. tissue	*Plat*	-1.04(p = 0.34)	1.08 (p = 0.13)
plasminogen activator. urokinase	*Plau*	1.03(p = 0.81)	1.01(p = 0.88)
plasminogen	*Plg*	-1.4(p≤0.05)	-1(p = 0.63)
**serine (or cysteine) peptidase inhibitor clade E member 1**	***Serpine1***	**-1.70(p≤0.01)**	-1.18(p = 0.31)
**serine (or cysteine) peptidase inhibitor clade H member 1**	***Serpinh1***	***1*.*89(p≤0*.*001)***	-1.07(p = 0.31)
tissue inhibitor of metalloproteinase 1	*Timp1*	-1.19 (p≤0.05)	-1.42(p = 0.14)
**tissue inhibitor of metalloproteinase 2**	***Timp2***	***-1*.*73(p≤0*.*01)***	-1.34 (p≤0.001)
**tissue inhibitor of metalloproteinase 3**	***Timp3***	***-1*.*74(p≤0*.*05)***	-1.36(p = 0.07)
tissue inhibitor of metalloproteinase 4	*Timp4*	-1.19(p = 0.20)	-1.16(p≤0.05)
integrin alpha 1	*Itga1*	1.43 (p≤0.05)	-1.25(p≤0.05)
integrin alpha 2	*Itga2*	-1.01(p = 0.42)	-1.06(p = 0.32)
**integrin alpha 2b**	***Itga2b***	***-1*.*49(p≤0*.*01)***	1.08(p = 0.10)
integrin alpha 3	*Itga3*	1.11(p = 0.34)	1.04(p = 0.06)
integrin alpha V	*Itgav*	1.08(p = 0.26)	1.09(p = 0.47)
integrin beta 1 (fibronectin receptor beta)	*Itgb1*	1.02(p = 0.70)	1.09(p = 0.44)
integrin beta 3	*Itgb3*	1.21(p = 0.17)	1.03(p≤0.05)
integrin beta 5	*Itgb5*	1.08(p≤0.05)	-1.42(p≤0.05)
integrin beta 6	*Itgb6*	1.46(p≤0.001)	1.19(p = 0.14)
integrin beta 8	*Itgb8*	1.07(p = 0.48)	-1.02(p = 0.68)
ae binding protein 1	*Aebp1*	-1.2(p≤0.001)	-1.87(p = 0.07)
annexin A4	*Anxa4*	1.04(p = 0.12)	-1.54(p = 0.06)
collagen type XI alpha 1	*Col4a1*	1.06(p = 0.17)	-1.09(p = 0.53)
**collagen type I alpha 1**	***Col1a1***	1.12(p = 0.27)	***-1*.*6(p≤0*.*05)***
collagen type III alpha 1	*Col3a1*	1.06(p = 0.06)	1.08(p = 0.31)
decorin	*Dcn*	*-1*.*23(p≤0*.*01)*	*-1*.*42(p≤0*.*05)*
dystrophin muscular dystrophy	*Dmd*	1.17(p≤0.05)	-1.2(p≤0.05)
coagulation factor II (thrombin) receptor	*F2r*	1.22(p = 0.43)	-1.23(p = 0.42)
**fibronectin 1**	***Fn1***	***-1*.*81(p≤0*.*01)***	-1.21(p = 0.07)
**aquaporin 4**	***Aqp4***	***-2*.*53(p≤0*.*05)***	-1.22(p≤0.05)
**phosphoenolpyruvate carboxykinase 1 cytosolic**	***Pdk1***	***-2*.*82(p≤0*.*01)***	**2.66*(p≤0*.*01)***

## Discussion

Our results show that the IGF-1 deficiency is associated with a reduction in heart contractibility, expressed as the quotient dD/dt (Fig 1), and insensibility to angiotensin II (Fig 2) with interstitial and perivascular fibrosis (Fig 4). In addition, we observed a dramatic expression reduction in genes coding for proteins involved in calcium dynamics and function as well as regulatory myosin proteins, α2 actin and natriuretic peptides (Fig 6). On the other hand, a 10 day-IGF-1 replacement therapy returned hemodynamic values and fibrosis to normality, albeit not all genes returned to normal values.

One of the most relevant aspects of this work is the presence of fibrosis in hearts not receiving exogenous insult, with the only exception of the partial IGF-1 deficiency. And secondly, that such fibrosis reverted when treated with IGF-1 for only 10 days. Although these findings are difficult to explain, some facts shed light over them. First of all, previous work using this same experimental model found that the single partial IGF-1 deficiency was associated with an altered structure of several organs, such as the brain [26], liver [25], and testicle [30]. Furthermore, the present study shows that hearts belonging to non-treated IGF-1-deficient mice overexpress *Serpine1* and *Serpinh1*, being sensitive to IGF-1 replacement therapy (Fig 7). It is known that *Serpine1*, also called PAI-1, is associated with alterations in extracellular matrix stability [31,32] and also seems to play role in early states of remodeling after damage [33]. *Serpine1* has also been found overexpressed in arterial hypertension and diabetes [34]. In addition *Serpinh1*, also called Hsp 47, has been identified as being a collagen chaperone [35] expressed under stress conditions [36]. Reduced expression of *Serpinh1* after tissue damage is associated with improvement of heart function [37]. Hence, the overexpression under IGF-1 deficiency may partly explain fibrosis.

In the same line, IGF-1-deficient mice displayed genes *Timp3* and *Timp2* significantly hypo-expressed. IGF-1 therapy was able to revert them to similar values to those found in controls (Fig 7). *Timp3* expression is relevant in the heart, as already reported [38]. It was shown that animals *Timp-3*^*(-/-)*^ after Ang II stimulation, show interstitial fibrosis without hypertrophy.

Another aspect that deserves particular mention is the aforementioned insensibility to Ang II observed in perfused hearts from the Hz group (Fig 2). Untreated Hz mice did not respond to Ang II, in fact, no vasoconstriction was observed at Ang II increasing concentrations. In the same line, untreated Hz mice showed a dramatic reduction of *Acta2* expression, which codes for α2actin, a contractile protein of the vascular smooth muscle. Such under-expression may explain the absence of response to Ang II. Accordingly, a significant increase of α2actin was observed in hearts from IGF-1 treated Hz mice (Hz+IGF-1) (Fig 6), possibly accounting for the increased response to Ang II in that same group. Mutations in this gene have been reported for causing aneurism and rupture of the aorta [39], as well as in coronary artery disease and stroke [40]. Other mutations in *Acta2* behaved as a multi-systemic syndrome with cerebral vascular disease and pulmonary hypertension [41].

Recent studies reveal non-canonical pathways for IGF1R in the heart which suggest that G_αi_ proteins also become activated, ultimately leading to [42–45] increases in cyclic adenosine monophosphate (cAMP), and hence to nuclear Ca^2+^ oscillations. This suggests another plausible mechanism for the reduced contractility observed, owing to a decreased Ca^2+^ tone caused by IGF-1 deficiency. Consistent with this, insensibility to Ang II could also, in part, be explained by this. It is widely known that intracellular signaling pathways cross-talk and convergence is really important. Also, recall that the angiotensin receptor AT1 (the most expressed in cardiomyocytes) mainly activates G_αi_ proteins, alike IGF1R in cardiomyocytes. Thus, IGF-1 deficiency might mitigate such signal in smooth muscle cells now lacking G_αi_ signal strength.

In addition, mice with partial IGF-1 deficiency (Hz group), showed under-expression of the synuclein alpha gene (*Snca*), whereas treated IGF-1 mice presented values similar to those in controls (Fig 6). Mutations in synuclein alpha have been linked to one type of inherited Parkinson’s Disease. Moreover, when mutations in this protein are present, cardiac sympathetic denervation ensues [46,47]. It is still not clear the exact role for this protein. Although some functions have been attributed to it, they are all unspecific. The one exception present in all studies is tyrosine hydroxylase activity. Tyrosine hydroxylase is responsible for the formation of noradrenalin, and aggregation of synuclein alpha [46]. Under this scenario, even though a perfused I/R model does not represent *in vivo* innervation, a chronic deprivation from sympathetic stimulation may have rendered the organ unresponsive to a series of events.

Regarding response to I/R, it would appear that IGF-1 deficiency, besides reducing basal contractility, renders the organ insensible to a normal physiological response to I/R. If carefully observed Fig 1 and Table 1, following I/R there is a marked reduction in contractility and HR within controls and treated animals, as it would be expected. However, untreated IGF-1-deficient animals do not show any variation in HR and contractility after I/R. An explanation could be that, because the IGF-1 deficient mice exhibit decreased basal contractility, the demand for oxygen is not as high as their counterparts. Consequently, being less affected by oxygen deprivation.

It has been proposed that, in mice cardiomyocytes, the overexpression of IGF1 produces physiological hypertrophy by protein translation [48] rather than pathologically altering gene expression like noradrenalin or Ang II. Parallel to this fact, *Igf1r* deletion resulted in normal heart growth although resistant to exercise-induced hypertrophy [49]. This is in accordance with findings disclosed in this work, where no change in absolute heart weight was observed despite the abrupt reduction in body weight. Then again, cardiac mass might not be properly structured, i.e. not as a result of a proper physiological hypertrophy, as suggested by others [48,49]. This may suggest that elevated relative cardiac weight in untreated Hz mice could be due to an excess of fibrotic mass or unorganized extracellular matrix. Fibrosis renders the organ with less functional and contractile mass, contributing to the decreased contractility observed. Also, the increment in interstitial space hinders the diffusion of nutrients and factors, making them partially unavailable, manifesting as a reduction in contractility.

Phospholamban (PLB) and sarcolipin (SLN) are two proteins that regulate the cardiac sarcoplasmic reticulum Ca^2+^ATPase (SERCA2a) [50]. SERCA2a is central in maintaining correct intracellular Ca^2+^ following contraction through active Ca^2+^ transport into the sarcolemma [51]. Thus, restoring the intracellular Ca^2+^ needed for the next contraction cycle [52]. Although the physiological relevance of SLN in the heart was only recently identified, overexpression of SLN reduced the apparent Ca^2+^ affinity for SERCA2a. *In vivo* assays of heart function showed a significant decrease in contractility [50]. Our results do not necessarily show the opposite, because overexpression of SLN could also inhibit SERCA, depleting sarcolemmal Ca^2+^ reserves and hence no new contraction cycle could effectively begin, affecting cardiac contractility. However, herein we link down-regulated SLN to reduced contractility, which, in our opinion, also makes sense. An increased affinity of SERCA for Ca^2+^ would mean an excessively rapid clearing of Ca^2+^ from the sarcomeres. Thus, reducing the Ca^2+^ tone and, with it, muscle force, consistent with a phenotype of reduced cardiac contractility. What is more, the mere IGF-1 deficiency is also associated to an under-expression of genes encoding proteins implicated in calcium dynamics such as s100 calcium BPA9 (*S100a9*) and s100 calcium BPA8 (*S100a8*), which could also be hijacking effective calcium dynamics and concomitant contraction.

As well, significant downregulation of the aquaporin 4 (*Aqp4*) gene (Fig 7G) in both Hz groups was observed. Even though the impact of this finding cannot be elucidated by results the present work, it is interesting to mention that *Aqp4*^*(-/-)*^ mice exhibit abnormalities in proteins related to calcium management [53] in the heart. Also, Alpha 2-Heremans Schmid Glycoprotein (*Ahsg*) gene was found underexpressed in both Hz groups, which seem to have calcification activities and related to inflammation [54]. Others [55] have found in *Ahsg*^*(-/-)*^ mice a greater concentration in pro-fibrogenic factors, augmented fibrosis, and tolerance to ischemia. All of which are consistent with findings in the present study. Moreover, expression of genes involved in the regulation and transport of myosin were also found underexpressed. It remains unknown whether these findings have relevance in heart physiology, nonetheless it might be affecting myosin function and thus contributing cardiac muscle contraction.

Co-cultured cardiac progenitor cells expressing c-Kit marker (receptor expressed in progenitor cells) have shown to secrete IGF-1, which improved cardiomyocyte survival and contractility [56]. Also, it has been discovered that fibroblasts subjected to physical stretching produce larger quantities of IGF1, including a rise in mRNA expression of auricular natriuretic peptide in ventricular myocytes [57], both of which seem to possess a beneficial action in the heart. Consistent with this, we observe a down-regulation of natriuretic peptides A and B, together with its receptor 3 in IGF-1 deficient animals. In the absence of these, the heart is less able to signal hemodynamic stress and therefore being subjected to it. It is demonstrated that IGF-1 replacement therapy did not reverse such downregulation, this may be explained by the fact that these IGF-1 secreting cells (progenitor cells fibroblasts) in the heart express a particular isoform of IGF-1 different from the circulating one [58], and that this isoform exerted protective effects after drug-induced infarct by upregulating sirtuin 1. Now, because the IGF-1 gene has been systemically truncated, these cells are unable to synthesize IGF-1 (any isoform derived from alternative splicing of the *Igf1* gene), and the rhIGF-1 isoform used for the treatment may not effectively activating the same signals that confer that physiological IGF-1 protection.

## Conclusions

In conclusion, data in this work show that IGF-1 partial deficiency is associated to a reduction in contractility and angiotensin II sensitivity, interstitial fibrosis as well as altered expression pattern of genes involved in extracellular matrix proteins, calcium dynamics, and cardiac structure and function. Certainly, these results are descriptive, they however provide a clear insight of the impact that IGF-1 partial deficiency on the heart. As well, this experimental model is suitable for studying cardiac disease mechanisms and exploring therapeutic options for patients under IGF-1 deficiency conditions such as ageing, metabolic syndrome, advanced cirrhosis.
